# Infective respiratory syncytial virus is present in human cord blood samples and most prevalent during winter months

**DOI:** 10.1371/journal.pone.0173738

**Published:** 2017-04-24

**Authors:** Angela Mary Fonceca, Abha Chopra, Avram Levy, Paul Stanton Noakes, Matthew Wee-Peng Poh, Natasha Leanne Bear, Susan Prescott, Mark Lloyd Everard

**Affiliations:** 1 School of Paediatrics and Child Health, University of Western Australia, Subiaco, Western Australia Australia; 2 Institute of Immunity and Infectious Diseases (IIID), Murdoch University, Murdoch, Western Australia, Australia; 3 PathWest Laboratory Medicine WA, Queen Elizabeth II Medical Centre, Nedlands, Western Australia, Australia; 4 School of Pathology and Laboratory Medicine University of Western Australia, Queen Elizabeth II Medical Centre, Nedlands, Western Australia, Australia; 5 Telethon Kids Institute, Subiaco, Western Australia, Australia; 6 Child and adolescent health service (CAHS), Department of Health, Subiaco, Western Australia, Australia; Kliniken der Stadt Köln gGmbH, GERMANY

## Abstract

**Background:**

Human respiratory syncytial virus (RSV) remains the most common cause of severe lower respiratory tract disease amongst infants, and continues to cause annual epidemics of respiratory disease every winter worldwide. Demonstrating placental transmission of viable RSV in human samples is a major paradigm shift in respiratory routes considered likely for RSV transmission.

**Methods:**

Droplet digital PCR (ddPCR) was used to identify RSV present in cord blood mononucleocytes (CBM). CBMs testing positive for RSV were treated with phytohemagglutinin (PHA), PHA and nitric oxide (NO) or PHA, NO and palivizumab, and co-cultured with HeLa cell monolayers. Subsequent immuno-staining for RSV was used to visualize infective viral plaques.

**Results:**

RSV was detected in 26 of 45 samples (57.7%) by ddPCR. CBM’s collected in winter were more likely to test positive for RSV (17/21 samples, risk = 80%, OR = 7.08; 95% CI 1.80–27.80; p = 0.005) compared to non-winter months (9/24 samples, 37.5%). RSV plaques were observed in non-treated and treated co-cultured HeLa monolayers.

**Conclusions:**

Demonstrating active RSV in CBMs suggests *in utero* transmission of infective virus to the fetus without causing overt disease. This is likely to have an important impact on immune development as well as future virus-host interactions, thereby warranting further investigation.

## Introduction

Despite more than 50 years of research, human respiratory syncytial virus (RSV) continues to cause annual epidemics of respiratory disease every winter worldwide [1]. RSV remains the most common cause of severe lower respiratory tract disease amongst infants and is also responsible for a significant burden of respiratory disease in the elderly [2–4]. As a member of the paramyxovirus family, RSV is an RNA virus related to other respiratory viruses such as influenza and parainfluenza [1].

RSV invades the epithelial cells of the lower respiratory tract following exposure to inhaled infective aerosolized droplets, or self-inoculation of the eyes and/or nose by contaminated fingers [2, 3]. The virus spreads from cell to cell by inducing cell fusion and the formation of syncytia [4]. This process leads to blocked airways and difficult breathing as a result of inflammation, cell death and raised mucus secretion [5]. In the absence of an effective vaccine or anti-viral therapy, treatment of RSV infection in infants is largely supportive and may include administration of supplemental oxygen, mechanical ventilation and fluid replacement [5, 6]. The failure to develop an effective vaccine is surprising given that there are only two subtypes of RSV which do not exhibit major antigenic drift [7]. This suggests improved insight into virus–host interactions are needed to progress treatment strategies.

RSV has also been shown to infect myeloid cells such as macrophages, dendritic cells (DCs), and cord blood monocytes [5–10]; however, a role for these alternate infection sites in RSV mediated airway disease is not currently well addressed. Furthermore, a paucity of data exists to explain ongoing evidence for RSV persistence observed beyond the epidemic periods in monocytes from human respiratory samples [11–13] and animal models [14–17]. Even more intriguing are observations to suggest RSV remains co-localised with cultured DCs for prolonged periods, with replication reactivation occurring spontaneously or following exposure to nitric oxide (NO) [5]. Together these studies point to monocyte infection by RSV, with a portion of these cells able to carry the virus for prolonged periods without causing obvious signs of infection; and the possibility of viral replication reactivation when triggered by appropriate stimuli.

Based on observations from studies which have shown RSV persistence and replication reactivation in cells of monocyte lineage [5–9], we hypothesized a presence of RSV in cord blood mononucleocytes (CBM) which could be activated by external stimuli. We sought to find evidence of RSV in CBMs to demonstrate human *in utero* transmission, and activation of live virus in these cells to suggest a cellular reservoir. Findings from this study could be used to explain the prevalence of RSV bronchiolitis observed in infants every winter and inform novel targets for effective treatment of RSV induced airway disease.

## Methods

### Patient samples

Cord blood samples collected from 45 term infants (23 female) from healthy mothers (18–45 years) recruited antenatally. Samples were collected from August 2002 until September 2003. Cord blood was collected immediately after delivery by means of venipuncture of placental vessels, as previously described [18]. Mononuclear cells were isolated by means of Ficoll-Hypaque gradient centrifugation (Lymphoprep; StemCell, Vancouver, Canada) and cryopreserved.

RNA purified from a pooled preparation of nasopharyngeal aspirates collected from patients diagnosed with RSV bronchiolitis were used as positive controls for RSVA and RSVB detection in cord blood samples by digital PCR analysis.

### Ethics statement

De-identified cord and maternal blood samples used for this study were collected under institutional ethics approval with informed adult written consent (Approval 728/EP, Child and Adolescent Health (CAHS) Human Research Ethics Committee) to investigate developmental immunology. De-identified nasopharyngeal aspirates collected from patients testing positive for RSV were obtained with agreement from PathWest Laboratory Medicine (Nedlands, Australia) to validate the presence of RSV by droplet digital PCR. According to the Human Tissue Acts of Australia, ethics approval was not required for use of these human samples as they were collected for therapeutic and/or diagnostic purposes and subsequently de-identified for pathology purposes, including assay validation [19]. All research investigation in this study was conducted according to the principles expressed in the Declaration of Helsinki.

### RSV detection

#### a) Droplet digital PCR (ddPCR)

RSV gene expression was measured using a commercially available fluorescent probe real-time PCR kit designed to detect all species of RSV (RSVSpp; GeneSig, Southampton, UK) on a droplet digital PCR platform (Bio-Rad, Hercules, US). Droplet digital PCR (ddPCR) is a next generation digital PCR with greater analytical sensitivity compared to conventional real-time PCR due to an enhanced ability to read nucleic acid at a single molecule level [20]. Viral RNA was extracted from thawed CBM cell pellets (Roche, Basel, Switzerland) and a minimum of 200ug used as template for cDNA synthesis using random primers and M-MLV reverse transcriptase (Promega, Southampton, UK). Twenty microliters of ddPCR fluorescent probe supermix with cDNA was added to the droplet generator cartridge and placed in the droplet generator with 70μl of generator oil. The resulting picoliter droplet emulsions were transferred to a 96 well PCR plate and cycled under the following conditions: 10 min hold at 98°C, 40 cycles of 95°C for 30s then 60°C for 60s and finally a 10 min hold at 98°C. After amplification the plate was transferred to the droplet reader to measure the number of positive and negative droplets based on fluorescence amplitude. The number of template molecules per microliter of starting material was estimated by QuantaSoft™ ddPCR software (Bio-Rad, Hercules, US) using an internal Poisson algorithm described previously [20].

#### b) Immunostaining for RSV plaque detection

Immuno-probing using an anti-RSV antibody was used to detect RSV plaques in order to demonstrate infective RSV detected by ddPCR in monocyte precursor cells contained in cord blood samples. Briefly, cryopreserved cord blood mononucleocytes (CBM) were thawed and allowed to rest for 3hrs at 37°C/5% CO2 in specialised dendritic cell media, X-VIVO (Lonza, Basel, Switzerland). Following this, cultures were treated with one of the following: phytohaemagglutinin (PHA, 1ug/ml; Sigma-Aldrich, St Louis, US); PHA with a soluble nitric oxide donor (NO, 65ug/ml; S-Nitroso-N-acetyl-DL-penicillamine, Sigma-Aldrich, St Louis, USA); or PHA with NO and palivizumab (PZB, 1600ug/ml; Synagis® MedImmune; Gaithersburg, US). All cultures were incubated for 24hrs and then added to HeLa monolayers (American Tissue Culture Collection) grown to 70% confluence in Dulbecco’s minimum essential medium (DMEM) supplemented with fetal calf serum (10% v/v). After 48 hours culture supernatants were removed and the remaining HeLa cell monolayers washed with PBS, and fixed with methanol containing hydrogen peroxide (1% v/v). These fixed HeLa cultures were then probed for RSV expression using anti-RSV HRP conjugated monoclonal antibody (Bio-Rad, Hercules, US) followed by incubation with Sigma-Fast Red (DAB; Sigma-Aldrich, St Louis, US) to detect the presence of RSV as described previously [21]. Stained RSV plaques were counted for analysis.

### Statistical analysis

A total of 45 samples were assessed for RSV expression and a minimum of 11 samples were used to compare RSV plaque assay formation in each treatment group. All samples were run in duplicate and all experiments completed at least twice to ensure reproducibility. Odd’s ratios and relative risk for RSV detected in cord samples were calculated using cross-tabulations with Fishers exact analysis. Due to an absence of data regarding the prevalence of RSV in CBMs, post-hoc power analysis was used to assess if the study interpretations were feasible based on the sample size available. The observed power was calculated at 82% based on the relative risks of RSV detection and sample size used. In order to mitigate concerns surrounding use of observed power and demonstrate the precision of these findings, confidence intervals are reported for the effect sizes calculated. Due to the small sample size and skewed distribution of the dataset, Mann Whitney-U analysis was used to compare RSV expression between birth seasons, and to assess differences in RSV plaque numbers between non-treated and treated co-cultured cells. Stata software by StataCorp (College Station, Texas, USA) was used to complete graphs and statistical analysis for this study with significance taken as p = <0.05 for two-tailed tests. Graphs represent median values with interquartile ranges unless stated otherwise.

## Results

### RSV detection in cord blood

**Fig 1:**
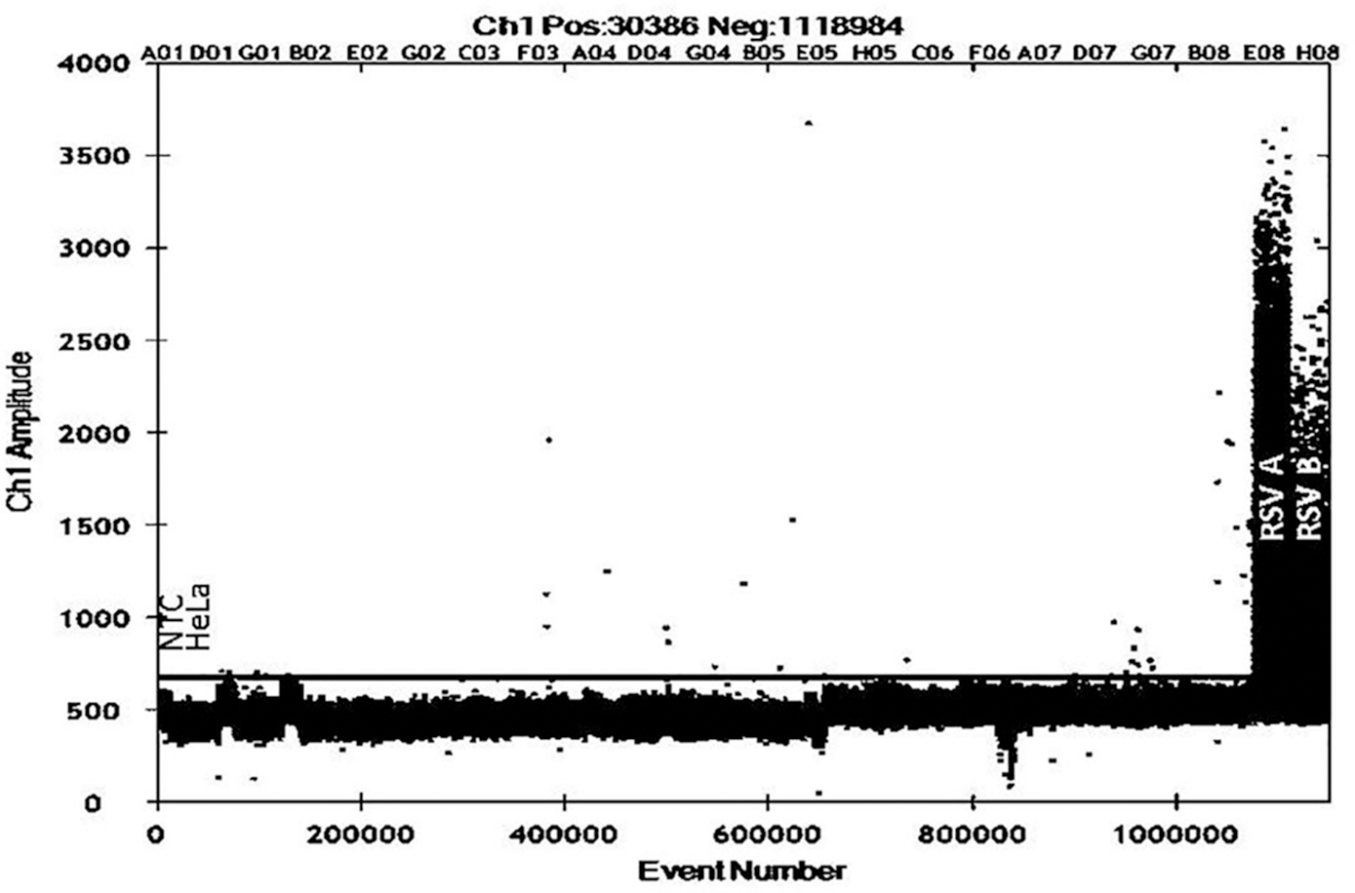
RSV N gene expression in cord blood samples measured by droplet digital real time PCR (ddPCR) using fluorescent probe detection. Cord blood samples were assessed for RSV N gene expression using a commercially available RSV fluorescent probe real-time PCR detection kit, with droplets prepared using BioRad ddPCR fluorescent probe immersion oil and droplet generator. RNA purified from a pooled preparation of nasopharyngeal aspirates collected from patients diagnosed with RSV bronchiolitis were used as positive controls for RSVA and RSVB detection. Non-template control and cDNA prepared from non-infected HeLa cells were used as negative controls to set the appropriate background expression threshold (black line). All samples were run in duplicate with events above the threshold line considered positive (26/45 samples, 57.7%).

Low level RSV expression was detected in human cord blood samples using droplet digital PCR (26/45 samples, 57.7%, Fig 1), suggesting *in utero* transmission of RSV to the human fetus. RSV could not be detected in a subset of 16 matched maternal bloods analysed by the same methods.

### Birth season distribution of cord blood samples testing positive for RSV

**Fig 2:**
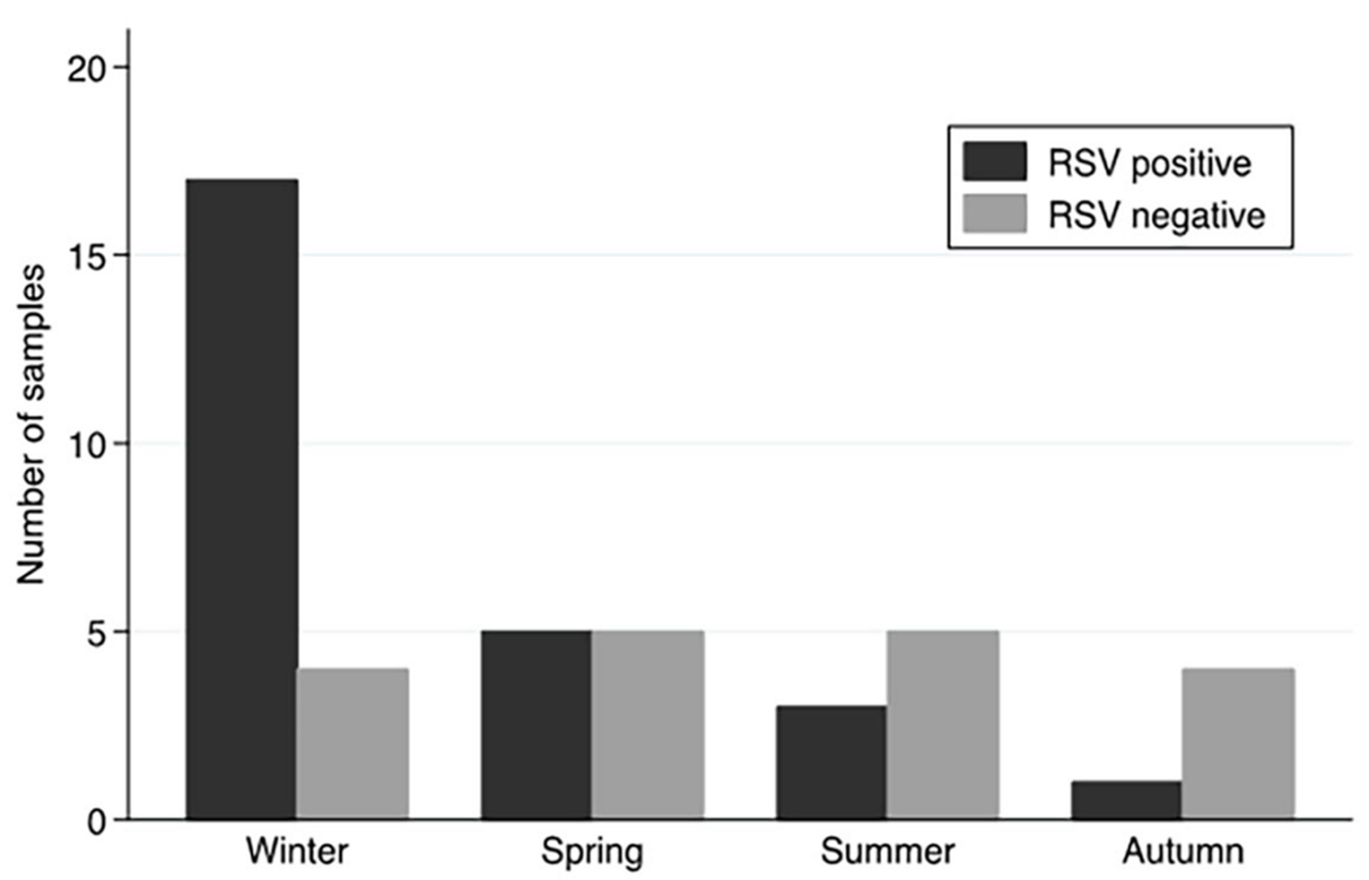
Birth season distribution for cord blood samples tested for RSV by ddPCR. Of the 45 samples tested for RSV N gene expression by ddPCR, the largest proportion of positive samples was observed in those collected during winter months (17/26).

**Fig 3:**
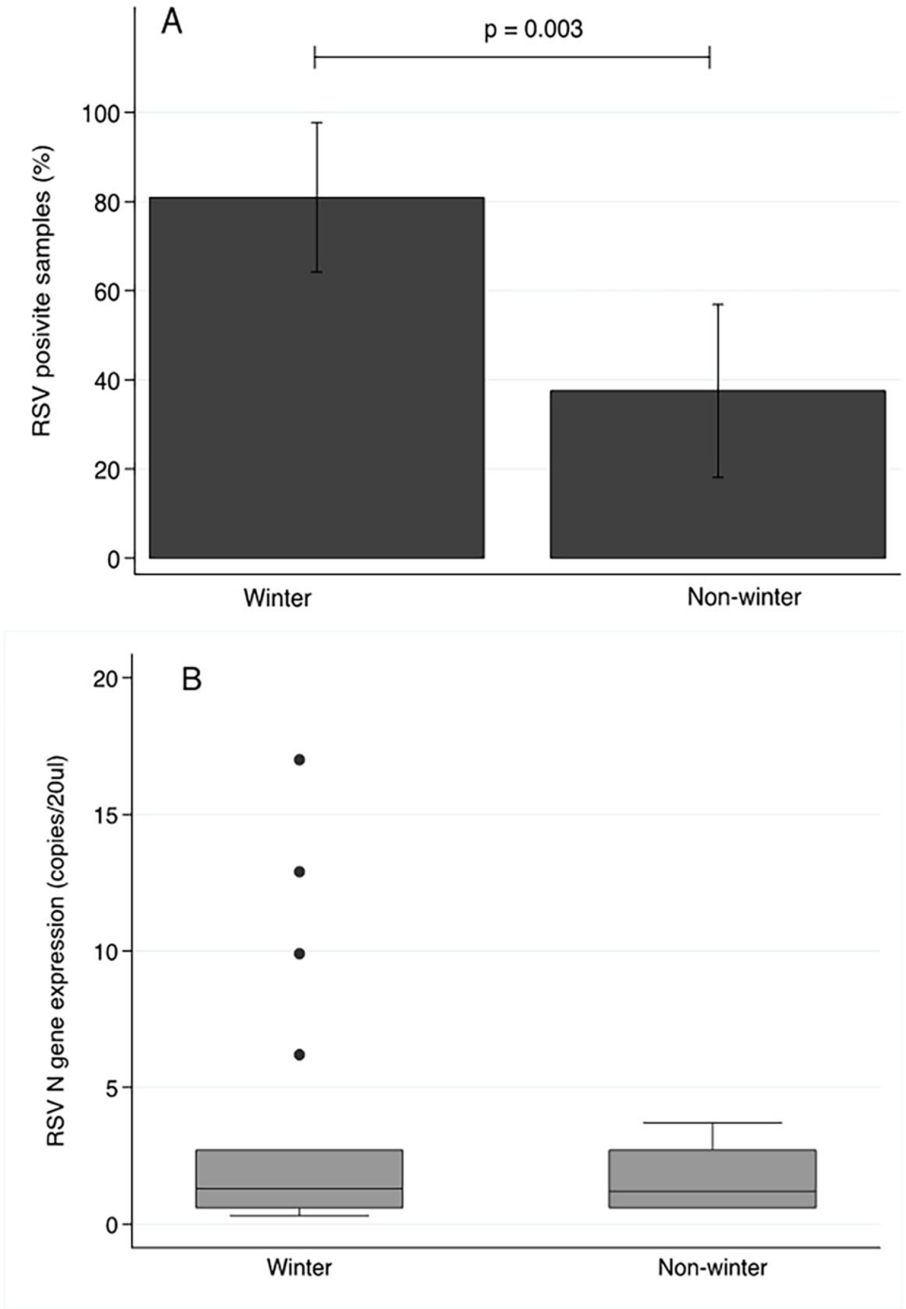
**Relative risk (A) and expression (B) of RSV N gene detected in cord blood samples collected in winter and non-winter months.** RSV N gene expression was more likely to be detected in samples collected during winter birth months (17/21, 81%; 95% CI 64.2–97.7) compared to cord blood samples from non-winter birth months (9/24, 37.5%; 95% CI 18.1–56.9, Fishers exact analysis). Of those samples where RSV was detected, no significant difference in RSV N gene expression was observed between winter and non-winter birth months (Mann-Whitney U analysis, p = 0.567).

Samples testing positive for RSV were identified across all birth seasons (Fig 2), with a greater number of RSV positive samples observed in winter (17/21) compared to non-winter birth months (9/24). A significantly raised odds ratio (OR = 7.08; 95% CI 1.80–27.80; p = 0.005) substantiated the significantly raised risk of RSV detection in cord bloods collected in winter (81%; 95% CI 64.2–97.7) compared to those collected in non-winter months (37.5%; 95% CI 18.1–56.9, p = 0.003; Fig 3A). While RSV was present in a greater proportion of samples collected in winter, the amount of virus was not significantly different to those samples testing positive and collected in non-winter months (Winter months: 1.3 copies/20ul IQR 0.6–2.7; Non winter months: 1.2 copies/20ul IQR 0.6–2.7; p = 0.567, Fig 3B).

### Cord blood co-cultures

**Fig 4:**
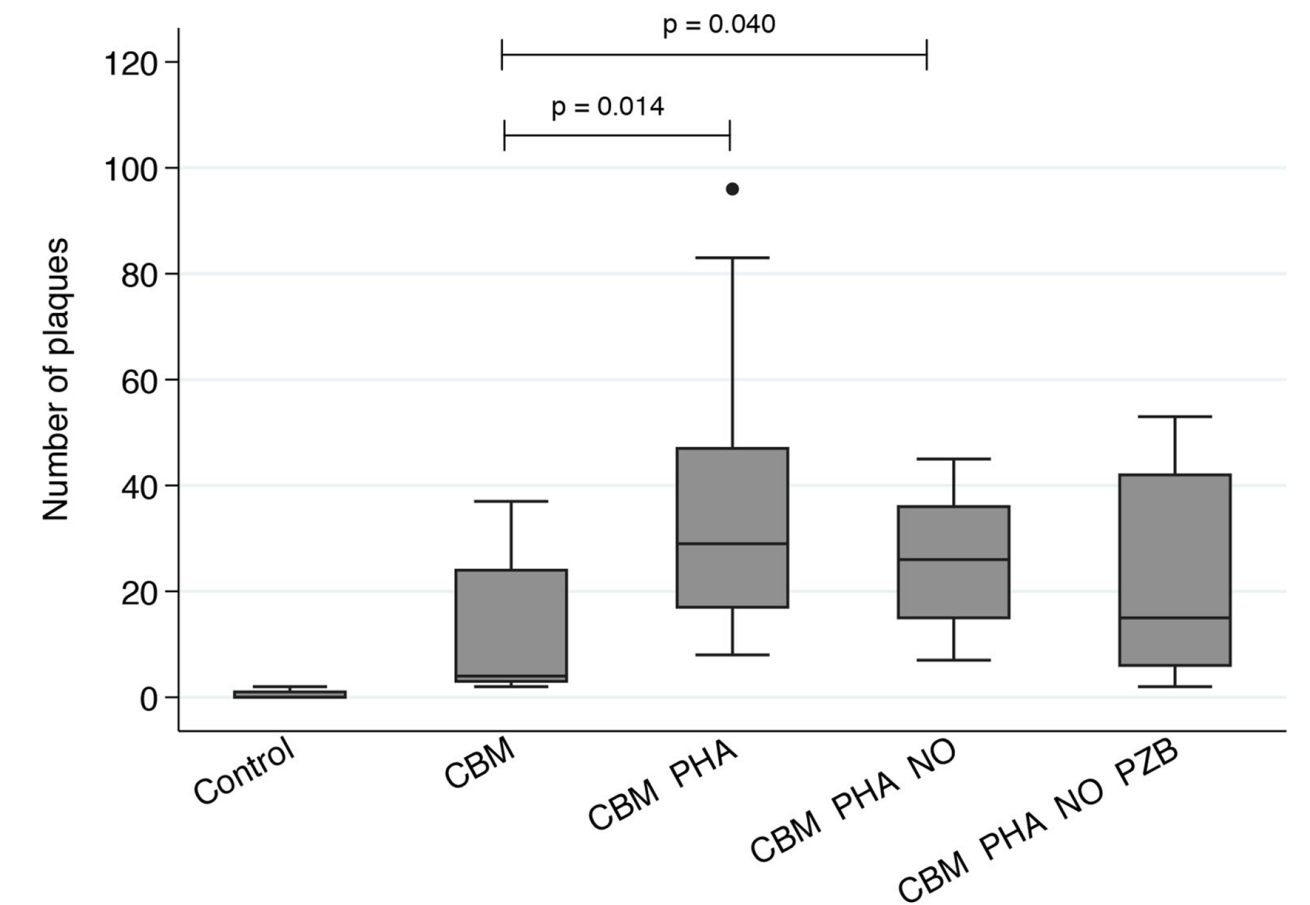
RSV plaques detected in HeLa cultures co-cultured with cord blood mononucleocytes (CBM) matured with PHA and treated with soluble nitic oxide (NO) and palivizumab (PZB). Cryopreserved cord blood samples were treated with PHA, ‘PHA with NO’ or ‘PHA with NO and PZB’ for 24hrs, then added to HeLa cells grown to 70% confluence. Significantly more RSV was detected in all HeLa monolayers co-cultured with CBMs regardless of treatment compared to HeLa monolayers alone (p<0.01). Non-treated CBMs co-cultured with HeLa cells had less RSV plaques then similar cultures treated with PHA and PHA with NO. While RSV was detected in co-cultures treated with ‘PHA, NO and PZB’ this was not significantly different to non-treated CBM co-cultures (Mann-Whitney U analysis, p = 0.193; n = 11 samples for all treatments).

Low level spontaneous release of RSV was observed in epithelial cells co-cultured with CBMs compared to HeLa monolayers alone (p<0.01, Fig 4). RSV release was significantly enhanced when CBMs were matured with phytohaemagglutinin (PHA, p = 0.014) and treated with nitric oxide (NO), used as an environmental trigger of RSV replication (p = 0.040). This effect was attenuated by treatment with the therapeutic anti-RSV antibody palivizumab (p = 0.193), confirming RSV release from these cells.

## Discussion

This is the first study to show evidence of low level RSV expression in human cord blood samples, suggesting *in utero* transmission of RSV to the human fetus. Importantly, release of infective RSV from cultured cord blood mononucleocytes indicates these cells harbor active virus. While RSV was present in a greater proportion of samples collected in winter (OR = 7.08), the amount of virus was not significantly different to those samples testing positive and collected in non-winter months.

We believe acute maternal respiratory infection acquired during annual RSV winter epidemics in late pregnancy may explain the greater number CBM samples testing positive for RSV in winter. RSV infections are common throughout adult life but generally cause mild symptoms in healthy adults, meaning presentations of RSV induced illness in healthy adults are unusual [22]. Therefore, it is not surprising that prospective data regarding the mother’s respiratory health during pregnancy was not available to further support transmission of active virus from a maternal RSV infection. Interestingly RSV could not be detected in a subset of matched maternal bloods analysed by the same methods.

Of the non-winter seasons RSV was most prevalent in spring. This may represent persistence following acquisition of RSV during winter months. DCs have been shown to harbor RSV [17] with spontaneous viral release being more pronounced when treated with environmental factors associated with winter months [5]. If the same applied to maternal sites of RSV persistence, under appropriate circumstances spontaneous release could result in RSV transmission and detection in CBMs in non-winter months [5, 23]. To date, only two RNA non-retroviruses, hepatitis C and human Pegivirus, are known to successfully persist in human hosts with normal and otherwise healthy immune systems [24]. Both viruses are transmitted by blood, sexually and from mother to child [24]. While a number of immune evasion mechanisms have been identified for these RNA viruses, the host mechanisms permitting persistence remain to be fully elucidated and may be relevant to DC-bound persistent RSV [24, 25].

Low level spontaneous release of RSV observed in epithelial cells co-cultured with CBMs suggest the fetus is exposed to low levels of infective RSV *in utero*. RSV release was significantly enhanced when CBMs were matured with phytohaemagglutinin (PHA) and treated with nitric oxide (NO), used as an environmental trigger of RSV replication. This effect was attenuated by treatment with the therapeutic anti-RSV antibody palivizumab, confirming RSV release from these cells. Therefore, this data suggests RSV is able to transmit from and between cells of monocyte lineage without inducing inflammatory symptoms usually associated with infection.

Importantly, exposure to infective RSV *in utero* during fetal immune system development may cause RSV immune tolerance [26, 27] explaining weak RSV antibody responses observed in infancy and adult life [28–30]. Diminished immune memory responses contribute to poor herd immunity, a factor considered to underpin annual epidemics of RSV induced respiratory disease observed every year worldwide [1, 3, 30]. Immune tolerance to RSV would also explain why RSV vaccines have failed to generate suitable antibody responses to date [26]. In light of these findings maternal immunization against RSV would seem a more effective option over post-natal vaccine strategies [31]. However, further investigation to determine RSV immune tolerance as a result of *in utero* exposure and the impact of viral exposure during fetal development is warranted due to maturing antigen-presenting precursor cell populations over the course of gestation [32].

As this study was conducted retrospectively it was not possible to explore the impact of RSV detection on infant lung structure and function. An association between viral respiratory illness in asthmatic mothers and increased lower respiratory tract symptoms amongst their infants during the first year of life has been shown previously [33]. The authors speculate that maternal respiratory infection may influence immune responses such that the off-spring may be more susceptible to asthma [33]. Two retrospective studies have produced results that appear to support this suggestion [34, 35], however further investigation is needed to clarify the influence of viral infection on these observations.

The novel data generated in this study indicates RSV can cross the placenta and infect the fetus without causing overt disease. Trans-placental transmission of RSV appeared to be most common during the winter epidemic but was not limited to this period. While the implications of these findings remain confined to understanding the dynamics of RSV transmission, it is likely the effects of *in utero* infection on host immune responses will have an important role in developing effective RSV vaccines and treatment in the future.
